# Stability of Cadmium Passivation in Weakly Alkaline Soil: Impact of Material, Dosage and Plant Cultivar

**DOI:** 10.3390/toxics14060508

**Published:** 2026-06-11

**Authors:** Jinpeng Yu, Yan Zhang, Hui Wang, Hong Pan, Quangang Yang, Yuping Zhuge, Yanhong Lou

**Affiliations:** National Engineering Research Center for Efficient Utilization of Soil and Fertilizer Resources, College of Resources and Environment, Shandong Agricultural University, Daizong Road, Tai’an 271018, China; yujinpengsd@163.com (J.Y.);

**Keywords:** cadmium contamination, passivation stability, application dosage, wheat cultivar, phosphate rock, bentonite

## Abstract

Passivation is a feasible approach for remediating heavy metal-contaminated soil. However, how passivation stability depends on material type, dosage, and plant cultivar remains unclear. Phosphate rock powder (PR) and bentonite (BN) were applied for passivation at three dosages, and the passivation activity was evaluated on high- and low-Cd-accumulating wheat cultivars. Phosphate rock powder (PR) and BN decreased the available Cd content in the soil by 11.52–26.65% and 11.08–35.00%, and in the wheat grains by 7.28–49.94% and 14.14–57.61%, respectively. PR_3_ and BN_3_ enhanced wheat yield by 12.77–21.31% and 12.23–18.67%, respectively. The passivation activity of both materials increased with increasing dosage. The optimal ranges for effective, stable Cd passivation were 0.29–0.61 and 3.85–8.46 t ha^−^^1^ for PR and BN, respectively. Path analysis revealed that PR acts mainly through increases in soil available phosphorus and associated changes in Cd fractions, whereas BN acts primarily through the soil cation exchange capacity; grain Cd was chiefly associated with reactive Cd fractions. The different Cd accumulation capacities of wheat cultivars affected the passivation effects of PR and BN. The soil of Jimai22 showed significantly lower EXC-Cd and Carb-Cd and significantly higher FeMnOz-Cd than Zhoumai18. Moreover, soil pH was higher for Jimai22 than for Zhoumai18. These results suggest that combining the selection of suitable passivation materials, optimising the dosage and planting low-Cd-accumulating cultivars is an effective strategy for maintaining Cd passivation in weakly alkaline soils.

## 1. Introduction

Soil is a crucial foundation for human survival and development; however, heavy metal (e.g., cadmium [Cd]) pollution due to sewage irrigation, excessive fertilisation and industrial waste discharge has raised widespread concerns, especially regarding arable land [1]. In China, approximately 150 million mu (1 mu = ~667 m^2^) of arable land has been contaminated with heavy metals, affecting 10 million tonnes of grain annually and causing economic losses of up to 20 billion yuan [2]. Excessive heavy metal accumulation in soil poses a serious threat, not only to soil and plants, but also to human health, as heavy metals, such as Cd, are readily absorbed by plants and subsequently accumulate in the human body through the food chain [3]. Soil Cd pollution is a widespread concern, and soil remediation and safety are critical for modern agriculture and sustainable land use [4]. Passivation is an effective method of remediating heavy metal–contaminated soil, especially in the major grain-producing areas of China. The underlying mechanism of passivation involves adsorption, precipitation, oxidation, reduction and complexation, which alter the form and activity of heavy metals, converting them into inactive, plant-unavailable forms [5]. Due to China’s large population, huge food demand and the shortage of high-quality arable land, passivation is the favoured technology in wheat cultivation areas, especially in soil with light-to-moderate heavy metal contamination [6]. Many studies have demonstrated that passivation materials exhibit good performance in remediating Cd-contaminated soil; however, studies focusing on their long-term stability are lacking [5].

The stability of passivation is especially important for ensuring the sustainable remediation of heavy metal–contaminated soil. Although many materials can effectively reduce Cd bioavailability in the short term, their stabilisation performance often declines over time because of complex soil–plant interactions [7]. Passivation is affected by various environmental conditions, such as soil pH, redox potential (Eh) and microbial activity, which, in turn, alter the speciation and mobility of heavy metals [8]. Yao et al. [9] reported that lowering the pH promotes the migration and release of Cd. Biswash et al. [10] reported that under waterlogged conditions, a decrease in soil Eh reduces the available Cd content. Zhou et al. [11] demonstrated that nitrogen fertiliser application in paddy soil induces rice roots to secrete organic acids, dissolving Cd precipitates and thereby enhancing Cd remobilisation in the soil.

The type and dosage of passivation materials also significantly affect the stability of Cd passivation. The long-term capacity of various materials for Cd passivation largely depends on differences between their surface functional groups, charge properties and mineral composition [12]. The iron oxide–modified biochar forms stable Fe–Cd co-precipitates in soil [13], whereas phosphorus-containing materials promote the formation of a stable Cd phosphate precipitate [14], exhibiting high passivation persistence. Alternatively, bentonite (BN) and biochar exhibit strong initial adsorption capacity, but their long-term stability is easily affected by changes in soil environmental conditions [5]. In addition, the application dosage directly affects reaction equilibria and the contact efficiency between materials and Cd^2+^. Moderate dosage can enhance Cd passivation efficiency, whereas excessive dosage may induce soil alkalisation or nutrient imbalance, ultimately inhibiting plant growth [15]. Therefore, studying the mechanism underlying passivation stability is crucial for developing durable and ecologically sound soil remediation strategies.

Different crop cultivars exert varying effects on the long-term stability of Cd passivation through distinct root exudates and rhizosphere pH regulation. Yang et al. [16] demonstrated that following biochar application, low-Cd-accumulating wheat cultivars sustain lower diethylenetriaminepentaacetic acid (DTPA)-Cd concentrations in the rhizosphere compared to high-Cd-accumulating cultivars. However, Ge et al. [17] reported that wheat root activity induces heightened soil microbial activity and increases organic acid release, leading to Cd reactivation in the soil. These studies demonstrate that wheat cultivars govern Cd uptake and concurrently modulate rhizosphere chemistry, thereby determining the persistence of passivation. Therefore, elucidating how different wheat cultivars affect passivation remediation of soil through rhizosphere interactions is crucial for enhancing the long-term stability of passivation remediation technology.

In this study, using two passivation materials (phosphate rock powder [PR] and BN at three application dosages) selected after preliminary research and two wheat cultivars (Jimai22 and Zhoumai18) with contrasting Cd accumulation capacities, a 3-year field trial was conducted on weakly alkaline Cd-contaminated soil. The objectives of this study were to (1) evaluate the passivation effects of PR and BN, (2) investigate the effect of the application dosage on passivation stability and (3) examine the impact of wheat cultivars with contrasting Cd accumulation capacities on passivation stability. The present findings will provide a crucial theoretical foundation and technical support for the remediation and safe use of weakly alkaline Cd-contaminated soil.

## 2. Materials and Methods

### 2.1. Experimental Site and Materials

The experimental site was located in farmland adjacent to a chemical plant in Liaocheng City, Shandong Province, China. At the time of the study, the average annual temperature was 13.3 °C; average annual precipitation, 558.7 mm; and average annual sunshine duration, 2213.2 h. In addition, the soil pH was 8.18; total Cd content, 0.83 mg kg^−1^; cation exchange capacity (CEC), 10.33 cmol kg^−1^; soil organic matter content, 12.41 g kg^−1^; soil total nitrogen (N), 1.46 g kg^−1^; available phosphorus (AP), 17.99 mg kg^−1^; and available potassium (AK), 256.67 mg kg^−1^.

PR was sourced from Jinan Rongguan Chemical Co., Ltd. (Jinan, Shandong, China), and the BN is sodium-based bentonite sourced from Shandong Huawei Bentonite Co., Ltd. (Jinan, Shandong, China). The pH, total phosphorus and particle size of PR were 8.42, 77.26 g kg^−1^ and 200 mesh, respectively. The pH, CEC and particle size for BN were 8.53, 90 cmol kg^−1^ and 200 mesh, respectively. The Cd content detected in PR and BN was almost nil (i.e., <0.01 mg kg^−1^).

Two wheat cultivars, Jimai22 (low Cd accumulation) and Zhoumai18 (high Cd accumulation) were selected [18]. Jimai22 was developed by the Shandong Academy of Agricultural Sciences (Jinan, Shandong, China), while Zhoumai18 was developed by the Zhoukou Academy of Agricultural Sciences (Zhoukou, Henan, China).

### 2.2. Experimental Design

Three application dosages (low, medium and high) were set for each passivation material (PR and BN); a dosage of 0 was used as the control (CK). The seven treatments were as follows: CK, no passivation material; PR_1_, 0.225 t ha^−1^ of PR; PR_2_, 0.450 t ha^−1^ of PR; PR_3_, 0.900 t ha^−1^ of PR; BN_1_, 3.0 t ha^−1^ of BN; BN_2_, 6.0 t ha^−1^ of BN; and BN_3_, 12.0 t ha^−1^ of BN. The plot area was 50.0 m^2^, and three replicates were used with a fully randomised design. Urea (46% N), diammonium phosphate (18% N and 46% P_2_O_5_) and potassium chloride (62% K_2_O) were used as basal fertilisers under a wheat–maize rotation system. Fertilisers were applied at a rate of 300 kg N ha^−1^, 180 kg P_2_O_5_ ha^−1^ and 90 kg K_2_O ha^−1^ during the wheat season and 195 kg N ha^−1^, 90 kg P_2_O_5_ ha^−1^ and 105 kg K_2_O ha^−1^ during the maize season. Passivation materials were applied three times: October 2018, June 2019 and October 2019. Fertilisers and passivation materials were evenly spread on the soil surface and rotary-tilled to a depth of 20 cm. Jimai22 was planted each year, and in October 2020, Zhoumai18 was planted on half of the area previously used for Jimai22. All field management was consistent in the 3 years of wheat planting.

### 2.3. Sample Collection

Soil and wheat grain samples were collected at maturity (June 2019, 2020 and 2021). Briefly, three crop rows in each plot were randomly selected and wheat was harvested in each row for 1 m continuously to determine yield and 1000-grain weight; whole wheat plants were carefully excavated, with entire root systems intact. The soil was gently removed by shaking the roots; any soil adhering to the roots was brushed off with a nylon brush and collected as the rhizosphere. A part of the fresh soil was stored at −20°C, while another portion was air-dried, passed through 18 and 100 mesh nylon sieves and analysed to determine the chemical properties and Cd fractions of soil. Whole wheat plants were brought back to the laboratory. The husk was removed, and the dried grains were ground into a fine powder to be used for determining heavy metal concentrations.

### 2.4. Measurement Indices

#### 2.4.1. Cd Content in Plants and Soil

DTPA-extractable Cd concentrations in soil were determined according to the GB/T 23739-2009 standard [19] using a diethylenetriaminepentaacetic acid (DTPA) extraction solution (0.005 mol L^−1^ DTPA, 0.01 mol L^−1^ CaCl_2_, 0.1 mol L^−1^ triethanolamine, pH 7.3) at a soil-to-solution ratio of 1:2 (*w*/*v*), with shaking for 2 h at 25 °C. Cd in plant samples was digested using a nitric acid-perchloric acid (HNO_3_-HClO_4_, 4:1, *v*/*v*) mixture based on the GB 5009.15-2014 standard [20]. The Cd fractions of soil were determined using the Tessier five-step sequential extraction method, which separates Cd into exchangeable (EXC-Cd), carbonate-bound (Carb-Cd), Fe-Mn oxide-bound (FeMnOz-Cd), organic matter-bound (OM-Cd) and residual (RES-Cd) fractions. Cd concentrations in all digested and extraction solutions were determined using inductively coupled plasma-optical emission spectroscopy (ICP-OES; iCAP 7200 spectrometer; Thermo Fisher Scientific, Waltham, MA, USA), as previously described [21].

#### 2.4.2. Chemical Indicators of Soil

Soil pH was measured using a pH metre (PHS-3C; Leici, Shanghai, China) at a water-to-soil ratio of 2.5:1 (*v*/*w*) after equilibration for 30 min. Soil cation exchange capacity (CEC) was determined by the ammonium acetate (NH_4_OAc) exchange method (1 mol L^−1^ NH_4_OAc, pH 7.0). Available phosphorus (AP) was extracted with 0.5 mol L^−1^ NaHCO_3_ (pH 8.5) and determined by the molybdenum antimony colorimetric method. Available potassium (AK) was extracted with 1 mol L^−1^ NH_4_OAc and determined by flame photometry [22].

#### 2.4.3. Passivation Stability Index and Optimal Application Dosage

The passivation stability index (PSI) was used to evaluate both the intensity and the temporal stability of Cd passivation under different PR and BN application dosages. For each indicator (DTPA-Cd, grain Cd, soil pH, soil AP and soil CEC), the PSI was used to integrate passivation efficiency and interannual stability as follows:

(1) $$\begin{array}{c} \mathrm{PSI}\;=\;\frac{1}{n}\sum_{i=1}^{n}\left[{\left(\frac{X_{\mathrm{CK},i}\;-{\;X}_{t,i}}{X_{\mathrm{CK},i}}\right)}^{\delta_{i}}{\left(\frac{X_{t,i\;}-\;X_{\mathrm{CK},i}}{X_{\mathrm{CK},i}}\right)}^{1-\delta_{i}}\right](1-CV_{i}) \end{array},$$

where *X*_t,_*_i_* and *X*_CK,_*_i_* are the mean values of indicator *i* in treatment and control arms, respectively; CV*_i_* = SD*_i_*/mean*_i_* represents the interannual variability; and *δ_i_* = 1 for indicators decreasing with effective passivation (DTPA-Cd and grain Cd content) or *δ_i_* = 0 for indicators increasing with effective passivation (soil pH, soil AP and soil CEC). The overall PSI was calculated as the mean of the five indicators (*n* = 5).

The relationship between the PSI and the PR/BN application dosage was examined using nonlinear regression by fitting a logistic model:

(2) $$\mathrm{PS}I\;=\;\frac{a}{1\;+\;e^{-b(R-c)}},$$

where *a*, *b* and *c* are fitted parameters. The optimal application dosage range was determined from the fitted curve by identifying *R*_opt_, defined as the inflection point of the logistic curve (maximum marginal gain), and *R*_95_, defined as the application dosage at which the PSI reached 95% of the asymptotic maximum. For this logistic model, *R*_opt_ was c and *R*_95_ was obtained analytically as $c\;+\;\frac{1}{b}\ln\left(19)\right.$.

### 2.5. Statistical Analysis

All data analyses were performed using Microsoft Excel 2016 (Redmond, WA, USA) and IBM SPSS 26.0 software (Armonk, NY, USA). One-way analysis of variance (ANOVA) was performed within each year to compare treatment effects, followed by Duncan’s multiple-range test for multiple mean comparisons. Interannual differences within each treatment were assessed by paired t-tests comparing the same experimental plots across years. Statistical significance was set at *p* < 0.05 for all tests. Path analysis and hierarchical clustering heatmap analysis were performed in R version 4.5.2 software (R Foundation for Statistical Computing, Vienna, Austria). All charts and graphs were created using Origin 2025 software (OriginLab Corporation, Northampton, MA, USA).

## 3. Results

### 3.1. Available Cd Content in Soil

**Figure 1:**
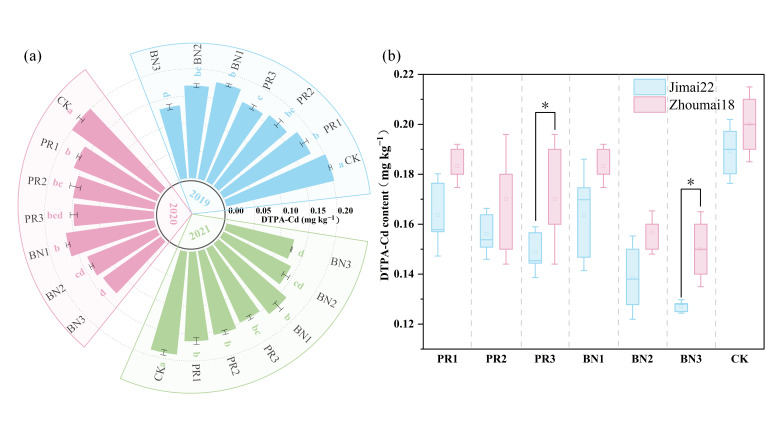
Effects of different passivation materials on the available DTPA-Cd content of soil. (**a**) Soil DTPA-Cd content from 2019 to 2021 and (**b**) soil DTPA-Cd content of different wheat cultivars after treatment. DTPA: diethylenetriaminepentaacetic acid. Different lowercase letters and asterisks above bars indicate significant differences among treatments (*p* < 0.05).

Compared to CK, all PR and BN dosages significantly reduced the soil DTPA-Cd content by 11.52–26.65% and 11.08–35.00%, respectively, over the 3-year dose-dependently (Figure 1a). Notably, the DTPA-Cd content was 10.68% lower with PR_3_ than with PR_1_ in 2019 but consistently lower with BN_3_ than with BN_1_ in all 3 years, with a reduction of 25.53%, 20.39% and 22.36% in 2019, 2020 and 2021, respectively. No significant annual variations in the soil DTPA-Cd content were observed over the 3 years regardless of the PR dosage. However, the soil DTPA-Cd content decreased by 16.29% and 15.33% in 2020 and 2021, respectively, with BN_2_ than with BN_1_. Furthermore, the soil DTPA-Cd content decreased by 20.86% in 2019 with BN_3_ than with BN_2_, with no significant differences observed in 2020 and 2021. Regarding annual variations, BN_2_ significantly reduced the soil DTPA-Cd content by 19.21% and 18.09% in 2020 and 2021, respectively, compared to 2019, while BN_1_ and BN_3_ caused no significant interannual variation. In addition, compared to the soil DTPA-Cd content of Jimai22, that of Zhoumai18 increased by 14.25% and 18.05% with PR_3_ and BN_3_, respectively (Figure 1b).

### 3.2. Cd Content in Wheat Grains

**Figure 2:**
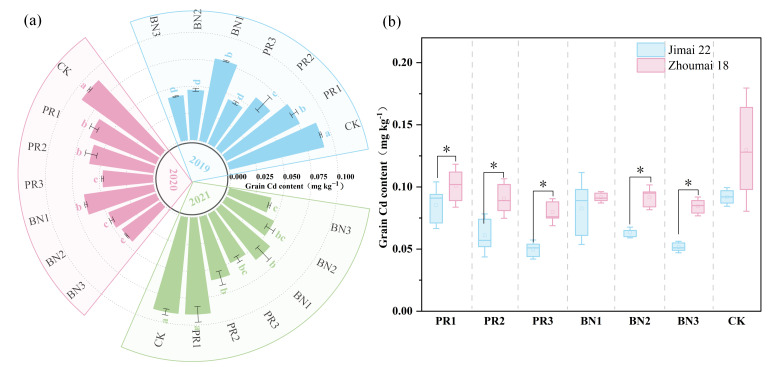
Effects of different passivation materials on the grain Cd content. (**a**) Grain Cd content from 2019 to 2021 and (**b**) grain Cd content of different wheat cultivars after treatment. Different lowercase letters and asterisks above bars indicate significant differences among treatments (*p* < 0.05).

Compared to CK, all PR and BN dosages (except PR_1_ in 2019) significantly reduced the grain Cd content by 7.28–49.94% and 14.14–57.61%, respectively, over the 3 years (Figure 2a). Notably, the grain Cd content was significantly lower with PR_3_ than with PR_1_ in all 3 years, with a reduction of 40.94%, 29.11% and 41.74% in 2019, 2020 and 2021, respectively. Similarly, the grain Cd content was significantly lower with BN_3_ than with BN_1_ over the same period, with a reduction of 45.95%, 32.16% and 38.39% in 2019, 2020 and 2021, respectively. Furthermore, the grain Cd content decreased by 21.38% and 28.48% in 2019 and 2021, respectively, with PR_2_ than with PR_1_, with no significant differences observed in 2020. Similarly, the grain Cd content decreased by 24.87% and 22.05% in 2019 and 2020, respectively, with PR_3_ than with PR_2_, with no significant differences observed in 2021. Regarding annual variations, PR_1_ significantly decreased the grain Cd content by 28.66% in 2020 compared to 2021, while PR_2_ and PR_3_ caused no significant interannual variation.

The grain Cd content decreased by 39.90% and 26.95% in 2019 and 2020, respectively, with BN_2_ than with BN_1_, with no significant differences observed in 2021. Regarding annual variations, BN_3_ significantly decreased the grain Cd content by 7.69% and 13.59% in 2019 and 2020, respectively, compared to 2021, while BN_1_ and BN_2_ caused no significant interannual variation. In addition, compared to the grain Cd content of Jimai22, that of Zhoumai18 increased by 18.36%, 48.63% and 60.40% with PR_1_, PR_2_ and PR_3_, respectively, and by 44.74% and 63.23% with BN_2_ and BN_3_, respectively (Figure 2b).

### 3.3. Physicochemical Properties of Soil

**Figure 3:**
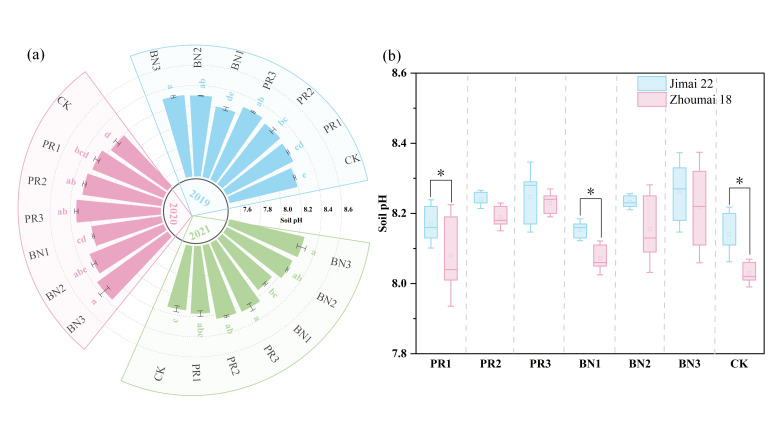

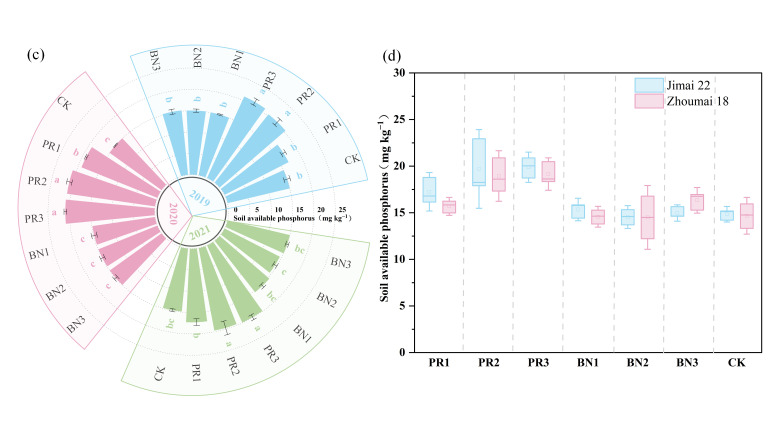

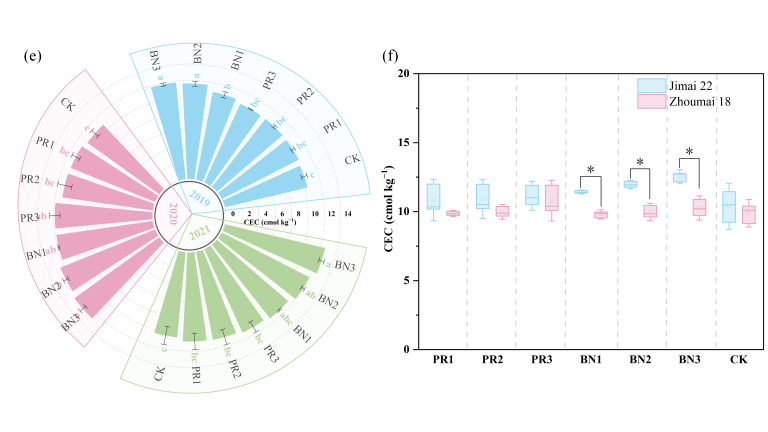

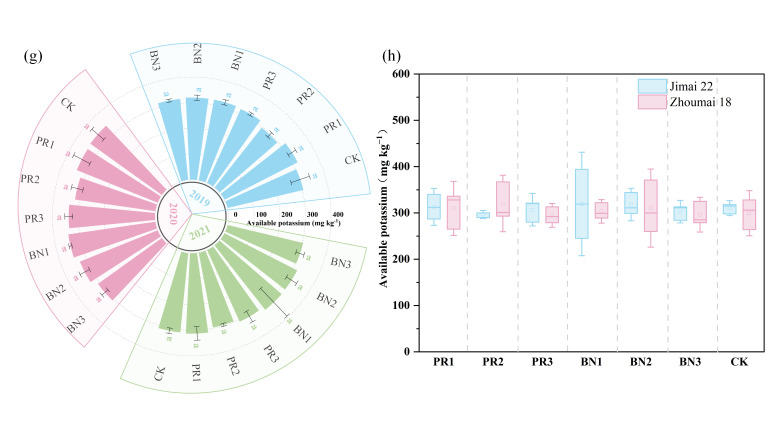
Effects of different passivation materials on soil pH, AP, CEC and AK. (**a**) Soil pH from 2019 to 2021, (**b**) soil pH of different wheat cultivars after treatment, (**c**) soil AP from 2019 to 2021, (**d**) soil AP of different wheat cultivars after treatment, (**e**) soil CEC from 2019 to 2021, (**f**) soil CEC of different wheat cultivars after treatment, (**g**) soil AK from 2019 to 2021 and (**h**) soil AK of different wheat cultivars after treatment. AK: available potassium; AP: available phosphorus; CEC: cation exchange capacity. Different lowercase letters and asterisks above bars indicate significant differences among treatments (*p* < 0.05).

Compared to CK, all PR and BN dosages (except PR_1_ in 2019 and 2021 and BN_1_ in all 3 years) significantly increased soil pH by 0.07–0.15 and 0.09–0.18 units, respectively (Figure 3a). Notably, soil pH was slightly higher (by 0.04 units) in 2019 with PR_3_ than with PR_1_, and no significant differences in soil pH were observed among other treatments within the 3-year period. Regarding annual variations, soil pH decreased significantly by 0.09 units from 2020 to 2021 with both PR_1_ and PR_3_, with no significant year-to-year differences observed for the other treatments. Furthermore, soil pH consistently increased more with BN_3_ than with BN_1_ in all 3 years, with a rise of 0.10, 0.17 and 0.11 units in 2019, 2020 and 2021, respectively. In addition, soil pH increased by 0.09 units in 2019 with BN_2_ than with BN_1_, with no significant differences observed between BN_2_ and BN_1_ in 2020 and 2021. Regarding annual variations, BN_1_ significantly reduced soil pH by 0.05 units in 2021 compared to 2019; similarly, BN_2_ significantly reduced soil pH by 0.05 and 0.06 units in 2021 compared to 2019 and 2020, respectively, while the other treatments caused no significant interannual variation. In addition, compared to the soil pH of Jimai22, that of Zhoumai18 significantly decreased by 0.11, 0.09 and 0.08 units with CK, PR_1_ and BN_1_, respectively (Figure 3b).

Compared to CK, all PR dosages (except PR_1_ in 2019 and 2021) significantly increased the soil AP (12.55–40.56%; Figure 3c). Notably, compared to PR_1_, PR_3_ and PR_2_ increased the soil AP by 21.16% and 24.88%, respectively, in 2019, with similar increases in 2020 (8.73% and 9.22%, respectively) and 2021 (14.21% and 15.27%, respectively); PR_3_ and PR_2_ caused no significant differences in the soil AP over the 3-year period. Regarding annual variations, PR_1_ significantly enhanced the soil AP by 13.93% in 2020 compared to 2019, while PR_2_ and PR_3_ caused no significant interannual variation (Figure 3d).

Compared to CK, all BN dosages (except BN_1_ in 2021) significantly increased the soil CEC by 5.13–20.82%, while PR_3_ significantly increased the soil CEC by 10.41% in 2021 (Figure 3e). Notably, in 2019, the soil CEC increased by 6.94% and 8.71% with BN_3_ and BN_2_, respectively, compared to BN_1_. Regarding annual variations, PR_3_, BN_1_ and BN_2_ significantly enhanced the soil CEC by 13.08%, 9.18% and 6.97%, respectively, in 2020 compared to 2019, and BN_3_ significantly enhanced the soil CEC by 7.66% and 5.83% in 2020 and 2021, respectively, compared to 2019. In addition, compared to the soil CEC of Jimai22, that of Zhoumai18 decreased by 16.66%, 20.03% and 22.23% with BN_1_, BN_2_ and BN_3_, respectively (Figure 3f).

However, no significant differences were observed in the soil AK among all treatments (Figure 3g,h).

### 3.4. Wheat Yield

**Figure 4:**
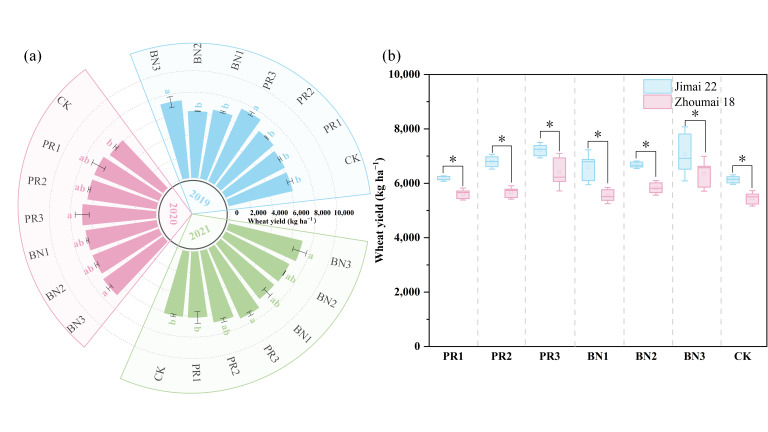
Effects of different passivation materials on wheat yield. (**a**) Wheat yield from 2019 to 2021 and (**b**) wheat yield of different cultivars after treatment. Different lowercase letters and asterisks above bars indicate significant differences among treatments (*p* < 0.05).

Compared to CK, PR_3_ and BN_3_ significantly increased the wheat yield by 12.77–21.31% and 12.23–18.67%, respectively, over the 3 years, while the remaining treatments did not exhibit this trend (Figure 4a). Notably, in 2019, the wheat yield with PR_3_ was 20.02% and 14.29% higher than with PR_1_ and PR_2_, respectively, and the wheat yield with BN_3_ was 11.39% and 16.69% higher than with BN_1_ and BN_2_, respectively. Regarding annual variation, PR_2_ and BN_2_ significantly enhanced the wheat yield by 4.34% and 7.06%, respectively, in 2021 compared to 2019. In addition, compared to the Zhoumai18 yield, the Jimai22 yield increased by 10.13–19.94% and 11.50–18.75% at all PR and BN dosages, respectively (Figure 4b).

### 3.5. Passivation Stability Index

**Figure 5:**
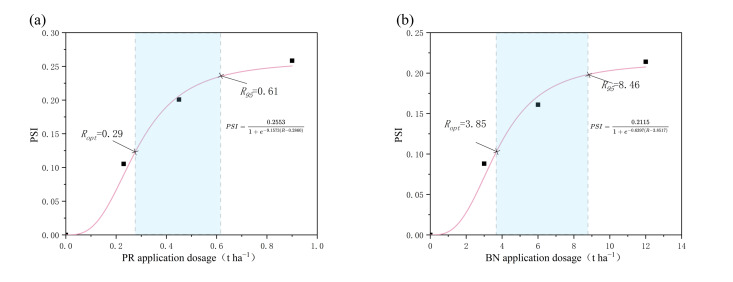
Relationship between (**a**) PR application dosage and PSI and (**b**) BN application dosage and PSI. PSI: passivation stability index.

All PR and BN dosages increased the PSI. In the case of PR treatment (Figure 5a), the PSI followed a logistic growth trend (*R*^2^ = 0.9876); *R*_opt_ = 0.29 t ha^−1^ and *R*_95_ = 0.61 t ha^−1^. Similarly, in the case of BN treatment (Figure 5b), the PSI followed a logistic growth trend (*R*^2^ = 0.9822); *R*_opt_ = 3.85 t ha^−1^ and *R*_95_ = 8.46 t ha^−1^. Overall, the PSI indicated that Cd passivation stability increased sharply with the application of a material with low-to-moderate passivation dosage, but the effect gradually plateaued at a higher passivation dosage. Therefore, the optimal ranges for effective and stable Cd passivation were found to be 0.29–0.61 t ha^−1^ for PR and 3.85–8.46 t ha^−1^ for BN.

### 3.6. Cd Fractions of Soil

**Figure 6:**
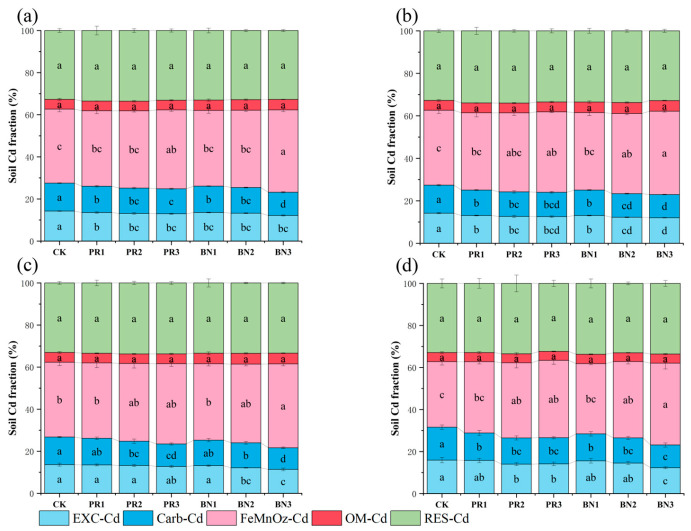
Effects of different passivation materials on soil Cd fractions of (**a**) Jimai22 in 2019, (**b**) Jimai22 in 2020, (**c**) Jimai22 in 2021 and (**d**) Zhoumai18 in 2021. EXC-Cd: exchangeable cadmium fraction; Carb-Cd: carbonate-bound cadmium fraction; FeMnOz-Cd: Fe–Mn oxide-bound cadmium fraction; OM-Cd: organic matter–bound cadmium fraction; RES-Cd: residual cadmium fraction. Different lowercase letters within the same color segment indicate significant differences among treatments for that Cd fraction (*p* < 0.05).

Compared to CK, all PR and BN dosages (except PR_1_, PR_2_, PR_3_ and BN_1_ in 2021) significantly reduced the soil EXC-Cd content by 5.01–11.54% and 4.82–17.09%, respectively, over the 3 years (Figure 6a–c). Notably, the soil EXC-Cd content decreased by 7.81% and 14.34% in 2020 and 2021, respectively, with BN_3_ compared to that with BN_1_ and by 6.36% in 2021 with BN_2_ compared to that with BN_1_. Regarding annual variation, BN_1_ and BN_2_ significantly reduced the soil EXC-Cd content by 3.71% and 7.53%, respectively, in 2020 compared to that in 2019. Similarly, BN_2_ and BN_3_ significantly reduced the soil EXC-Cd content by 8.74% and 6.95%, respectively, in 2021 compared to that in 2019. However, the remaining treatments showed no significant interannual variation.

Compared to CK, all PR and BN dosages (except PR_1_ and BN_1_ in 2021) significantly reduced the soil Carb-Cd content by 5.73–18.92% and 5.50–21.87%, respectively, over the 3 years. Notably, the soil Carb-Cd content decreased by 4.98% and 16.05% in 2019 and 2021, respectively, with PR_3_ compared to that with PR_1_. Regarding annual variation, PR_3_ and BN_3_ significantly reduced the soil Carb-Cd content by 7.24% and 6.09%, respectively, in 2021 compared to that in 2020 and by 10.60% and 7.25%, respectively, in 2021 compared to that in 2019. In addition, BN_2_ significantly reduced the soil Carb-Cd content by 8.69% in 2020 compared to that in 2019, while BN_1_ and BN_3_ showed no significant interannual variation.

Compared to CK, PR_3_ significantly enhanced the soil FeMnOz-Cd content by 6.59% and 7.53% in 2019 and 2020, respectively; BN_3_ significantly enhanced the soil FeMnOz-Cd content by 11.21%, 11.36% and 11.77% in 2019, 2020 and 2021, respectively; and BN_2_ enhanced the soil FeMnOz-Cd content by 7.09% in 2020. Notably, the soil FeMnOz-Cd content increased by 8.80%, 7.66% and 8.87% in 2019, 2020 and 2021, respectively, with BN_3_ than with BN_1_.

In addition, CK, PR_1_, PR_3_, BN_1_ and BN_2_ consistently increased the soil EXC-Cd content of Zhoumai18 by 16.76%, 16.91%, 11.03%, 18.08% and 21.62%, respectively, compared to that of Jimai22 (Figure 6d). In contrast, CK and PR_3_ significantly decreased the soil FeMnOz-Cd content of Zhoumai18 by 12.22% and 3.02%, respectively, compared to that of Jimai22. Moreover, BN_2_ and BN_3_ significantly increased the soil OM-Cd content of Jimai22 by 17.09% and 14.71%, respectively, compared to that of Zhoumai18.

### 3.7. Path Analysis

**Figure 7:**
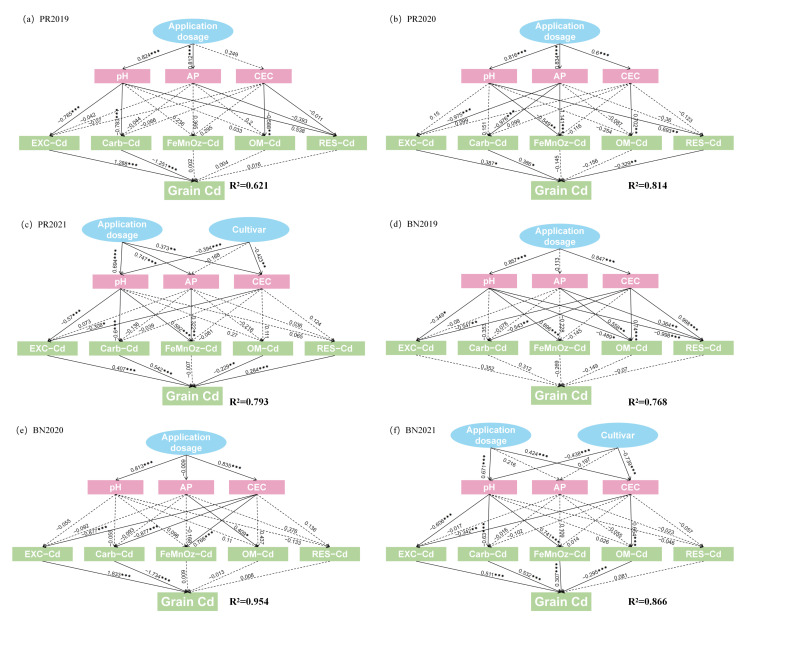
Path analysis of factors influencing Cd accumulation in wheat. Values adjacent to arrows are standardised path coefficients, and *R*^2^ indicates the proportion of variance explained for grain Cd in each model. Solid lines denote significant pathways, while dashed lines denote nonsignificant pathways. *** Significant at the 0.001 level; ** significant at the 0.01 level; * significant at the 0.05 level. (**a**) PR in 2019, (**b**) PR in 2020, (**c**) PR in 2021, (**d**) BN in 2019, (**e**) BN in 2020 and (**f**) BN in 2021. AP: available phosphorus; CEC: cation exchange capacity; EXC-Cd: exchangeable cadmium fraction; Carb-Cd: carbonate-bound cadmium fraction; FeMnOz-Cd: Fe–Mn oxide-bound cadmium fraction; OM-Cd: organic matter–bound cadmium fraction; RES-Cd: residual cadmium fraction.

Path analysis revealed pronounced interannual shifts in the dominant pathways linking application dosage, wheat cultivars, soil properties, Cd fractions and wheat Cd accumulation, with clear differences in both the direction and the relative strength of key paths between years. These year-to-year variations contrasted markedly between the PR and BN treatments across the 3 years (Figure 7):

• PR in 2019: Dosage had significant positive effects on pH (0.824) and AP (0.812). Soil pH showed a significant negative association with EXC-Cd (−0.785) and Carb-Cd (−0.783). Grain Cd was positively related to EXC-Cd (1.288) and negatively related to Carb-Cd (−1.251).
• PR in 2020: Dosage had significant positive effects on pH (0.816), AP (0.834) and the CEC (0.600). Soil pH showed a significant negative association with FeMnOz-Cd (−0.549) and a positive association with RES-Cd (0.693). Soil AP showed a significant negative association with EXC-Cd (−0.975) and Carb-Cd (−0.976) and a positive association with FeMnOz-Cd (1.141). Grain Cd was positively related to EXC-Cd (0.387) and Carb-Cd (0.386) and negatively related to RES-Cd (−0.329). The explained variance (*R*^2^) of grain Cd increased from 0.621 in 2019 to 0.814 in 2020.
• PR in 2021: These effects remained significant, although they reduced in strength (pH = 0.694; AP = 0.747), and were accompanied by a moderate positive effect on the CEC. Moreover, wheat cultivars had significant negative effects on pH (−0.394) and the CEC (−0.423). Soil pH showed a significant negative association with EXC-Cd (−0.57) and Carb-Cd (−0.6) and a positive association with FeMnOz-Cd (0.592). Soil AP showed a significant positive association with FeMnOz-Cd (0.322). The soil CEC showed a significant negative association with EXC-Cd (−0.308). Grain Cd continued to be positively associated with EXC-Cd (0.407) and Carb-Cd (0.542). In addition, a significant negative path from OM-Cd to grain Cd (−0.229) and a significant positive path from RES-Cd to grain Cd (0.284) were detected. The *R*^2^ of grain Cd remained high (0.793).
• BN in 2019: Dosage had significant positive effects on pH (0.857) and the CEC (0.847). The soil CEC showed a strong negative association with EXC-Cd (−0.547) and Carb-Cd (−0.543) and a positive association with OM-Cd (0.740) and RES-Cd (0.998). Soil pH showed a strong negative association with EXC-Cd (−0.349), OM-Cd (−0.489) and RES-Cd (−0.998) and a positive association with FeMnOz-Cd (0.896).
• BN in 2020: Dosage had significant positive effects on pH (0.813) and the CEC (0.835). The soil CEC showed a strong negative association with EXC-Cd (−0.877) and Carb-Cd (−0.877) and a positive association with FeMnOz-Cd (0.766). Grain Cd was positively related to EXC-Cd (1.833) and negatively related to Carb-Cd (−1.734). The *R*^2^ of grain Cd increased from 0.768 in 2019 to 0.954 in 2020.
• BN in 2021: These effects remained significant, although they reduced in strength (pH = 0.671; CEC = 0.424). Moreover, wheat cultivars had significant negative effects on pH (−0.438) and the CEC (−0.739). Soil pH showed a significant negative association with EXC-Cd (−0.606) and Carb-Cd (−0.63) and a positive association with FeMnOz-Cd (0.741). The soil CEC showed a significant positive association with OM-Cd (0.654) and a negative association with EXC-Cd (−0.342). Grain Cd was positively related to EXC-Cd (0.511), Carb-Cd (0.532) and FeMnOz-Cd (0.307) and negatively related to OM-Cd (−0.295). The *R*^2^ of grain Cd decreased from 0.954 in 2020 to 0.866 in 2021.

### 3.8. Hierarchical Clustering Heatmap Analysis

**Figure 8:**
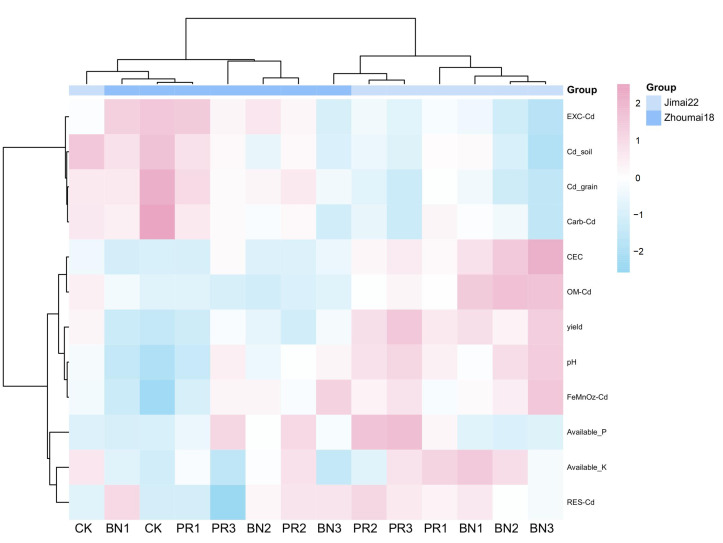
Comprehensive evaluation of passivation effects of PR and BN treatments applied to different wheat cultivars. Cd_soil: DTPA-Cd; CEC: cation exchange capacity; EXC-Cd: exchangeable cadmium fraction; Carb-Cd: carbonate-bound cadmium fraction; FeMnOz-Cd: Fe–Mn oxide-bound cadmium fraction; OM-Cd: organic matter–bound cadmium fraction; PR: phosphate rock powder; RES-Cd: residual cadmium fraction.

Hierarchical clustering heatmap analysis revealed treatment similarity (Figure 8). Cluster analysis grouped all treatments into four major clusters, and different treatments corresponding to different wheat cultivars were clearly divided into two major categories. Notably, the treatment corresponding to Jimai22 was grouped together with that corresponding to Zhoumai18 under BN_3_ processing. Each major category was further subdivided into four subcategories based on application dosage: (1) BN_1_, CK and PR_1_ applied to Zhoumai18 and CK applied to Jimai22; (2) PR_3_, BN_2_ and PR_2_ applied to Zhoumai18; (3) BN_3_ applied to Zhoumai18 and PR_2_ and PR_3_ applied to Jimai22; and (4) PR_1_, BN_1_, BN_2_ and BN_3_ applied to Jimai22. On the basis of the correlation heatmap of various indicators, BN_2_ and BN_3_ applied to Jimai22 were the optimal treatments. Cd fractions were categorised into two major groups: (1) EXC-Cd and Carb-Cd fractions, which negatively impacted passivation treatment, and (2) OM-Cd, FeMnOz-Cd and RES-Cd fractions, which positively impacted passivation treatment.

## 4. Discussion

### Principal Findings

In weakly alkaline soil, BN showed a markedly stronger Cd passivation effect than PR. BN treatment reduced soil DTPA-Cd and grain Cd content by 35.00% and 57.61%, respectively, while PR-induced reductions were only 26.65% and 49.94%, respectively. The passivation effect of PR relied on the precipitation reaction of AP in the soil to fix Cd; however, PR effectiveness was greatly affected by the soil pH. High-pH environments suppressed phosphorus release, limiting the passivation effect of PR. Li et al. [14] and Rezwan et al. [23] have reported similar results. In contrast, BN mainly enhanced the electrostatic adsorption capacity of Cd by increasing the soil CEC, and the passivation effect of BN was less affected by pH. Furthermore, the high specific surface area and permanent negative charge of BN provide stronger adsorption ability for Cd^2+^ [24]. Wang et al. [25] reported similar findings. Interannual variations showed that the mechanisms of transformation of soil Cd forms by both BN and PR changed significantly over time. After continuous PR application, the Cd transformation mechanism went through two stages: initially, the pH increase dominated, promoting the transformation of EXC-Cd to Carb-Cd, as pH elevation facilitated the formation of precipitates between Cd^2+^ and CO_3_^2−^; in the second year, with phosphorus accumulation, the transformation mechanism shifted to one dominated by AP, promoting further transformation of Carb-Cd to FeMnOz-Cd and RES-Cd. However, after the PR application ceased, the transformation of EXC-Cd to RES-Cd was no longer significant due to the reduction in phosphorus and the ageing of the soil system. The mechanism underlying BN treatment, however, showed a different evolutionary pattern. In the early stage of application, both pH and the CEC jointly dominated the transformation of EXC-Cd to Carb-Cd, FeMnOz-Cd, OM-Cd and RES-Cd. In the second year, after continuous BN application, as pH had no significant effect on the transformation of the Cd fraction, the Cd fixation mechanism gradually shifted to adsorption, dominated by the CEC. This is because BN’s pH-increasing capability is limited and short-lived, constrained by buffering effects in weakly alkaline soil, whereas the CEC, as an inherent property of BN, does not decay over time but, instead, strengthens due to material accumulation, ensuring its long-term stable passivation effect [26].

In addition, application dosage is a key factor determining passivation efficiency and long-term stability. PR_1_ exhibited poor passivation stability for soil Cd. This is because the passivation effect of PR depends on phosphorus release; without a continuous supply of soluble phosphorus, the passivation efficiency of PR declines sharply [27]. Conversely, BN treatments exhibited good passivation stability across all dosages. This is because BN has a high specific surface area and CEC, so its passivation effect is less affected by soil pH. This result is consistent with that of the study by Wang et al. [25], who reported that 4–8% clay can remove up to 70% of heavy metal pollutants, and a higher clay content provides more active sites, thereby enhancing adsorption efficiency. The passivation stability of both BN and PR increased with higher application dosage; however, when considering both cost and effectiveness, a higher dosage does not necessarily lead to proportionally greater benefits. The capacity of soil to immobilise Cd has an upper limit, constrained by both the availability of adsorption sites and the dissolution equilibrium in soil solution [28]. This finding is consistent with the conclusion drawn from our analysis following the establishment of an application dosage versus passivation stability model. Similar findings have been reported in studies on biochar, clay and lime as passivation materials [28,29,30]. Cd passivation management should aim to optimise rather than maximise application dosage.

Incorporation of the temporal dimension further revealed the dose–time response dynamics of BN and PR. Notably, after three consecutive applications, the cumulative input of the low-dose treatments (PR_1_ and BN_1_) exceeded that of the single medium-dose treatments (PR_2_ and BN_2_). However, interannual monitoring showed that grain Cd and soil-available Cd under PR_1_ and BN_1_ did not exhibit a significant decreasing trend over the 3-year period. This indicated that the long-term accumulation of low-dose amendments does not result in a substantial improvement in passivation efficiency. In contrast, a single application at a moderate dose significantly reduced grain Cd and soil-available Cd in the short term. Specifically, low-dose passivation materials do not reach the effective passivation threshold; in addition, because of the material ageing, it is difficult to significantly improve the passivation effect. This result is consistent with the study by Rahimi et al. [31] who reported that higher amendment dosages more effectively reduce Cd bioavailability in contaminated soil. In addition, Shang et al. [32] demonstrated that ageing processes can redistribute previously adsorbed heavy metals, thereby weakening long-term stabilisation effects. Therefore, to overcome response lag and the limitations imposed by material ageing, soil remediation strategies should not rely on the gradual accumulation of low-dose applications. Instead, amendments should be applied at the optimal threshold range during the initial stage of remediation (0.29–0.61 t ha^−1^ for PR and 3.85–8.46 t ha^−1^ for BN).

In addition to material properties and application dosage, biological disturbances due to differences in crop rhizosphere microenvironments should not be overlooked [33]. When a high-Cd-accumulating wheat cultivar was introduced, the previously established chemical passivation equilibrium showed clear vulnerability. Specifically, after planting the high-Cd-accumulating cultivar, the soil DTPA-Cd content and the highly active soil EXC-Cd and Carb-Cd fractions remained significantly higher than those observed for the low-Cd-accumulating cultivar, even in PR- and BN-treated soil. Hierarchical clustering heatmap analysis provided intuitive evidence for this phenomenon: samples subjected to the same amendment type and dosage did not cluster closely together but instead showed clear separation on the basis of the wheat cultivar, indicating that cultivar differences had become a key driving force reshaping the distribution of soil Cd fractions. Path analysis further revealed the mechanism underlying this biological disturbance. After the passivation material application ceased, cultivar effects markedly affected path coefficient tests, significantly altering the soil pH and CEC. These differences in rhizosphere effects may largely be attributed to the distinctive root exudation patterns of high-Cd-accumulating cultivars. The more active root systems of such cultivars may release specific organic acids or other compounds that can locally dissolve previously formed metastable precipitates in the rhizosphere microzone [34,35]. Zhu et al. [36] reported that dissolved organic matter derived from the rhizosphere of *Sedum alfredii* could significantly increase Cd mobility by forming soluble DOM–metal complexes through complexation and chelation of root exudates with Cd. Overall, the passivation stability depends not only on the properties and application dosage of passivation materials but also on the interaction between the passivation materials and the crop root system. Therefore, to safely use weakly alkaline soil with Cd contamination, appropriate passivation materials should be selected and their application dosage optimised, in combination with cultivating low-Cd-accumulating crop cultivars.

## 5. Conclusions

Bentonite (BN) had superior Cd passivation performance compared to PR in weakly alkaline, Cd-contaminated soil, and the optimal application dosages were 0.29–0.61 t ha^−1^ for PR and 3.85–8.46 t ha^−1^ for BN. Planting the wheat cultivar Jimai22 was more effective than planting Zhoumai18 in maintaining the stability of Cd passivation. Considering both economic cost and remediation efficiency, the optimal management strategy was to apply 3.85 t ha^−1^ of BN, combined with the low-Cd-accumulating cultivar Jimai22. Long-term monitoring is required to further verify the persistence and stability of passivation under field conditions. This study provides a practical strategy for maintaining passivation stability in weakly alkaline soils by integrating material type, dosage and plant cultivar, thereby supporting long-term soil remediation and food safety. However, although the 3-year field experiment provides valuable insights, long-term validation is required to fully assess passivation stability.

## Data Availability

The original contributions presented in this study are included in the article. Further inquiries can be directed to the corresponding authors.

## References

[1] Huang X., Li X., Zheng L., Zhang Y., Sun L., Feng Y., Du J., Lu X., Wang G. (2024). Comprehensive assessment of health and ecological risk of cadmium in agricultural soils across China: A tiered framework. J. Hazard. Mater..

[2] Qu Z., Zhang J., Zeng M., Zhang K., Xu D., Qi Y., Deng X. (2024). Impact of heavy metal hazard perceptions on pollution control intentions: Empirical evidence from rice farmers in China. J. Clean. Prod..

[3] Kumar P., Alhag S.K., Al-Shahari E.A., Al-Fakeh M.S., Abou Fayssal S., Bachheti R.K., Širić I., Eid E.M. (2025). Impact of irrigation with contaminated river water on growth, yield, and heavy metals accumulation in planted Armenian cucumber (*Cucumis melo* var. *flexuosus* (L.) Naudin.). Water Air Soil Pollut..

[4] Hou D., Jia X., Wang L., McGrath S.P., Zhu Y.-G., Hu Q., Zhao F.-J., Bank M.S., O’Connor D., Nriagu J. (2025). Global soil pollution by toxic metals threatens agriculture and human health. Science.

[5] Xing J.F., Cang L., Ren J.H. (2019). Remediation stability of in situ chemical immobilization of heavy metals contaminated soil: A review. Soils.

[6] Li Y., Li X., Kang X., Zhang J., Sun M., Yu J., Wang H., Pan H., Yang Q., Lou Y. (2023). Effects of a novel Cd passivation approach on soil Cd availability, plant uptake, and microbial activity in weakly alkaline soils. Ecotoxicol. Environ. Saf..

[7] Cui W., Li X., Duan W., Xie M., Dong X. (2023). Heavy metal stabilization remediation in polluted soils with stabilizing materials: A review. Environ. Geochem. Health.

[8] Zhang Y., Fu P., Li S., Deng W., Li S., Ni W., Zhang S. (2025). Freeze–thaw and dry–wet alternation regulate the impacts of Fe–C based passivator on soil heavy metals immobilization. J. Environ. Chem. Eng..

[9] Yao J., Qian J., Ji D. (2025). Machine learning-based analysis of heavy metal migration under acid rain: Insights from the RF and SVM algorithms. Minerals.

[10] Biswash M.R., Li K.-W., Xu R.-K., Uwiringiyimana E., Guan P., Lu H.-L., Li J.-Y., Jiang J., Hong Z.-N., Shi R.-Y. (2024). Alteration of soil pH induced by submerging/drainage and application of peanut straw biochar and its impact on Cd(II) availability in an acidic soil to indica-japonica rice varieties. Environ. Pollut..

[11] Zhou H.-Z., Wang B.-Q., Ma Y.-H., Sun Y.-Y., Zhou H.-L., Song Z., Zhao Y., Chen W., Min J., Li J.-W. (2025). The combination of metagenomics and metabolomics reveals the effect of nitrogen fertilizer application driving the remobilization of immobilization remediation cadmium and rhizosphere microbial succession in rice. J. Hazard. Mater..

[12] Jia Y., Li J., Zeng X., Zhang N., Wen J., Liu J., Jiku M.A.S., Wu C., Su S. (2022). The performance and mechanism of cadmium availability mitigation by biochars differ among soils with different pH: Hints for the reasonable choice of passivators. J. Environ. Manag..

[13] Si T., Yuan R., Qi Y., Zhang Y., Wang Y., Bian R., Liu X., Zhang X., Joseph S., Li L. (2024). Enhancing soil redox dynamics: Comparative effects of Fe-modified biochar (N–Fe and S–Fe) on Fe oxide transformation and Cd immobilization. Environ. Pollut..

[14] Li C., Tan X., Li X., Huang Y., Xiang C., Wu C., Guo J., Xue S. (2025). Simultaneous stabilization of cadmium and arsenic in soil by humic acid and mechanically activated phosphate rock. J. Hazard. Mater..

[15] Agbede T.M. (2025). Poultry manure improves soil properties and grain mineral composition, maize productivity and economic profitability. Sci. Rep..

[16] Yang S., Wu P., Jeyakumar P., Wang H., Zheng X., Liu W., Wang L., Li X., Ru S. (2022). Technical solutions for minimizing wheat grain cadmium: A field study in North China. Sci. Total Environ..

[17] Ge W., Gao F., Gao J., Ding J., Han L., Wang J., Zhang T., Li H., Yan Y. (2025). The synergistic impact of cadmium and the wheat rhizosphere on the soil bacterial community in alkaline cropland in Northern China. J. Soils Sediments.

[18] Ming Y., Zhang X., Yu H. (2018). The evaluation of Cd accumulation in grains of different wheat materials. Sci. Agric. Sin..

[19] (2009). Soil Quality—Determination of Available Lead and Cadmium in Soil—Atomic Absorption Spectrophotometry after DTPA Extraction.

[20] (2014). National Food Safety Standard—Determination of Cadmium in Foods.

[21] Sun S., Guan D., Xie Y., Tian F., Ji X., Wu J. (2025). Calcium–silicon–magnesium synergistic amendment enhances cadmium mitigation in *Oryza sativa* L. via soil immobilization and nutrient regulation dynamics. Agriculture.

[22] Lu W., Zhou Y., Ma X., Gao J., Guo J., Fan X., Xing W., Gao W., Lin M., Wang R. (2025). Impacts of organic fertilizer substitution on soil ecosystem functions: Synergistic effects of nutrients, enzyme activities, and microbial communities. Agronomy.

[23] Rezwan F., Kashem M.A., Hu H., Islam M.S. (2024). Mechanisms of metal immobilization in the soil by a phosphate compound with low molecular weight organic acids present. Commun. Soil Sci. Plant Anal..

[24] Xie X., Wu X., Shafi A., Dong D., Xu Y., Li Q., Hou S., Liu D., Xu W. (2024). Combined biochar and bentonite application for immobilization of cadmium and *Brassica chinensis* L. growth in contaminated soil. J. Soil Sci. Plant Nutr..

[25] Wang P., Shen X., Qiu S., Zhang L., Ma Y., Liang J. (2024). Clay-based materials for heavy metals adsorption: Mechanisms, advancements, and future prospects in environmental remediation. Crystals.

[26] Xu Y., Liang X., Xu Y., Qin X., Huang Q., Wang L., Sun Y. (2017). Remediation of Heavy Metal-Polluted Agricultural Soils Using Clay Minerals: A Review. Pedosphere.

[27] Maw T.T., Deng J., Li B., Zu Y., Li Z. (2025). Single super phosphate improves *Lolium perenne* quality and rhizosphere microorganism structure under combined cadmium and arsenic stress. Toxics.

[28] Zhang Y., Gao S., Jia H., Sun T., Zheng S., Wu S., Sun Y. (2024). Passivation remediation of weakly alkaline Cd-contaminated soils using combined treatments of biochar and sepiolite. Ecol. Process..

[29] Lan M.-M., Liu C., Liu S.-J., Qiu R.-L., Tang Y.-T. (2020). Phytostabilization of Cd and Pb in highly polluted farmland soils using ramie and amendments. Int. J. Environ. Res. Public Health.

[30] Sui F., Yang Y., Wu Y., Yan J., Fu H., Li C., Qin S., Wang L., Zhang W., Gao W. (2024). Cadmium minimization in grains of maize and wheat grown on smelting-impacted land ameliorated by limestone. Toxics.

[31] Rahimi M., Bertalan-Balázs B., Adelinia A., Ebrahimi E., Ojani M. (2024). Impact assessment of Zeolite, Ca-bentonite and Biochar amendments on Cd bioavailability and fractions in polluted calcareous soils. Environ. Earth Sci..

[32] Shang Z., Wang T., Ye Q., Wu P., Wu J., Sun L., Zhu N. (2024). An environmentally friendly strategy for reducing the environmental risks of heavy metals adsorbed by kaolinite. J. Environ. Manag..

[33] Sui F., Gao Z., Qiang C., Lu H., Ma J., Cui L., Quan G., Yan J. (2026). Mechanisms of rice rhizosphere response to soil amendments under heavy metal(loid) stress—A review. J. Soil Sci. Plant Nutr..

[34] Xiao Y., Guo W., Qi X., Hashem M.S., Wang D., Sun C. (2023). Differences in cadmium uptake and accumulation in seedlings of wheat varieties with low- and high-grain cadmium accumulation under different drought stresses. Plants.

[35] Yue M., Rao S., Liu X., Yang W., Yuan Y., Xu F., Cheng S. (2025). Cadmium hyperaccumulation in plants: Mechanistic insights and ecological implications. Phyton.

[36] Zhu X., Tu C., Zhou J., Yang S., Li Y., Wu L., Newman L.A., Luo Y. (2025). Cadmium phytoextraction by *Sedum alfredii* and *Sedum plumbizincicola*: Mechanisms, challenges and prospects. Int. J. Phytoremediat..

